# Single-Element 2D Materials beyond Graphene: Methods of Epitaxial Synthesis

**DOI:** 10.3390/nano12132221

**Published:** 2022-06-28

**Authors:** Kirill A. Lozovoy, Ihor I. Izhnin, Andrey P. Kokhanenko, Vladimir V. Dirko, Vladimir P. Vinarskiy, Alexander V. Voitsekhovskii, Olena I. Fitsych, Nataliya Yu. Akimenko

**Affiliations:** 1 Faculty of Radiophysics, National Research Tomsk State University, Lenin Av. 36, 634050 Tomsk, Russia; kokh@mail.tsu.ru (A.P.K.); vovenmir@gmail.com (V.V.D.); vinarskiy2017@gmail.com (V.P.V.); vav43@mail.tsu.ru (A.V.V.); 2 Scientific Research Company “Electron-Carat”, Stryjska St. 202, 79031 Lviv, Ukraine; i.izhnin@carat.electron.ua; 3 P. Sagaidachny National Army Academy, Gvardijska St. 32, 79012 Lviv, Ukraine; o.fitsych@ukr.net; 4 Department of Engineering Systems and Technosphere Safety, Pacific National University, Tihookeanskaya St. 136, 680035 Khabarovsk, Russia; n_akimenko@inbox.ru

**Keywords:** 2D materials, two-dimensional allotropes, graphene analogs, molecular beam epitaxy, borophene, aluminene, gallenene, indiene, thallene, silicene, germanene, stanene, plumbene, phosphorene, antimonene, bismuthene, selenene, tellurene

## Abstract

Today, two-dimensional materials are one of the key research topics for scientists around the world. Interest in 2D materials is not surprising because, thanks to their remarkable mechanical, thermal, electrical, magnetic, and optical properties, they promise to revolutionize electronics. The unique properties of graphene-like 2D materials give them the potential to create completely new types of devices for functional electronics, nanophotonics, and quantum technologies. This paper considers epitaxially grown two-dimensional allotropic modifications of single elements: graphene (C) and its analogs (transgraphenes) borophene (B), aluminene (Al), gallenene (Ga), indiene (In), thallene (Tl), silicene (Si), germanene (Ge), stanene (Sn), plumbene (Pb), phosphorene (P), arsenene (As), antimonene (Sb), bismuthene (Bi), selenene (Se), and tellurene (Te). The emphasis is put on their structural parameters and technological modes in the method of molecular beam epitaxy, which ensure the production of high-quality defect-free single-element two-dimensional structures of a large area for promising device applications.

## 1. Background

**Figure 1:**
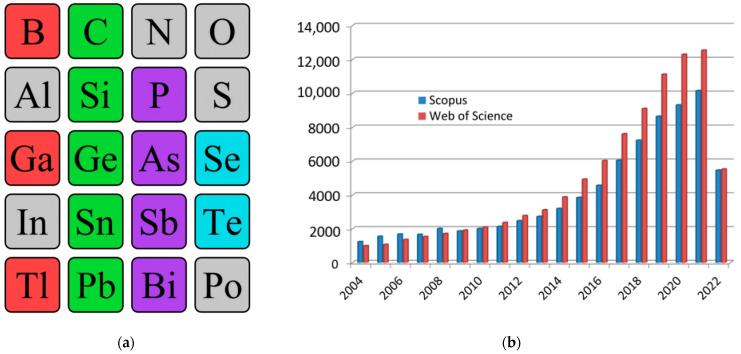
Single-element 2D materials (**a**) and number of publications with the keyword “2D material” in scientific analytical databases Scopus and Web of Science (**b**). In the excerption from the periodic table, synthesized single-element two-dimensional materials are highlighted: group IIIA—red, group IVA—green, group VA—violet, group VIA—blue.

Today, two-dimensional materials are one of the key research topics for scientists around the world [1,2,3,4,5]. Since 2004, when the first representative of a new class of materials, a monolayer of carbon atoms, was experimentally obtained in the work by Geim and Novoselov [6], we have seen a real boom in publications on this topic. Over the years, there has been a quantitative and qualitative leap both in the study of graphene itself and of other two-dimensional allotropes—*transgraphenes*, or *X-enes* (Figure 1a). As a characteristic numerical indicator confirming the above, we can cite data on the growth in the number of publications that include the keyword “2D material” in the international information and analytical systems of scientific citation Scopus and Web of Science (Figure 1b). By 2022, the number of publications crossed a milestone—more than 10,000 publications on the topic of 2D materials per year (30 articles per day or 1 article every hour).

This interest in two-dimensional materials is not surprising, since, thanks to their remarkable mechanical, thermal, electrical, magnetic, and optical properties, they promise to revolutionize electronics. Among the outstanding characteristics of 2D materials, the following can be distinguished: very high mobility of charge carriers, extreme strength of graphene due to strong carbon–carbon bonds, the ability to control energy structure and bandgap by changing the material composition, and a simple defect structure due only to the presence of vacancies and impurities. In addition, under certain conditions, exotic quantum effects can manifest in these materials: they can be topological insulators and high-temperature superconductors. The unique properties of 2D materials make them promising for the creation of completely new types of devices for functional electronics, nanophotonics, and quantum technologies [7,8,9,10,11,12].

Nowadays, the following existing two-dimensional allotropic modifications can be distinguished: graphene (C) and its analogs (*transgraphenes* or *X-enes*) borophene (B), aluminene (Al), gallenene (Ga), indiene (In), thallene (Tl), silicene (Si), germanene (Ge), stanene (Sn), plumbene (Pb), phosphorene (P), arsenene (As), antimonene (Sb), bismuthene (Bi), selenene (Se), and tellurene (Te) (Figure 1a).

They are very closely related to graphene and transgraphene derivatives functionalized by hydrogen ions (graphane (CH)*_n_*, silicane (SiH)*_n_*, germanane (GeH)*_n_*, and other *transgraphanes* (or *X-anes*) with the general formula (GH)*_n_*, where G is one of the elements that form the initial two-dimensional material: C, Si, Sn, Pb, B, P, etc.) or other ligands (for example, metal cations, hydroxyl groups, organic radicals, with the general formula (GR)*_n_*) [13].

Among the methods for creating two-dimensional materials, it is necessary to distinguish simple exfoliation techniques, chemical deposition methods, and molecular beam epitaxy. It is the latter method that makes it possible to obtain structures of the best quality: with low roughness, controlled thickness, and a high degree of uniformity over the substrate area. To achieve the desired quality, special attention must be paid to the selection of a suitable substrate and careful control of the deposition conditions, such as temperature and growth rate.

In this work, two-dimensional allotropes of various elements are considered. A review of the latest advances in growing single-element 2D materials by epitaxial methods is given. At the same time, the emphasis is put on their structural parameters and technological modes in the method of molecular beam epitaxy, which ensures the production of high-quality defect-free single-element two-dimensional structures of a large area, which are necessary for promising device applications.

## 2. Structural Characteristics of Single-Element 2D Materials

**Figure 2:**
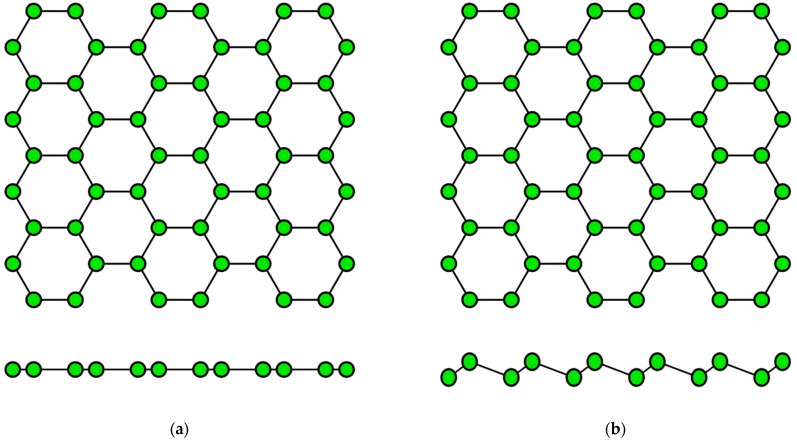
Honeycomb lattice of graphene (**a**) and graphene-like structure of two-dimensional materials of group IVA (**b**) (on this and following figures colored balls represent atoms and black lines represent interatomic bonds).

The concept of a two-dimensional allotropic modification of silicon was proposed in 1994 [14]. Subsequently, the new material was actively studied by theorists and was named “silicene” by analogy with graphene [15]. All graphene-like materials of elements of group IVA are characterized by mixed sp^2^–sp^3^ hybridization [16]. Unlike graphene (Figure 2a), the lattice of silicene, germanene, stanene, and plumbene is not absolutely flat, but curved. Their structure can be represented as two sublattices displaced relative to each other in the vertical direction (Figure 2b). Such curved honeycomb structure and ordered buckling of the surface lead to exceptional stability and increased flexibility compared with graphene. This geometry defines the wide spectrum of their potential applications in electronics [17,18,19]. Such a buckled hexagonal structure was also observed in all further works on the synthesis of transgraphenes of group IVA [20,21,22,23,24,25,26,27,28,29,30,31,32,33,34,35,36,37,38,39,40,41,42,43,44,45,46,47,48,49,50,51,52,53,54,55,56,57,58].

The data on the lattice parameters of group IVA graphene-like 2D materials (silicene, germanene, stanene, and plumbene) such as the distance *l* between nearest atoms in the honeycomb structure, the lattice constant *a*, and the displacement parameter between the upper and lower atoms δ are reviewed in Table 1.

Borophene, aluminene, gallenene, indiene, and thallene—representatives of group IIIA transgraphenes—can have various modifications of the crystal lattice [59,60,61,62,63,64,65,66,67,68,69,70,71,72,73].

**Figure 3:**
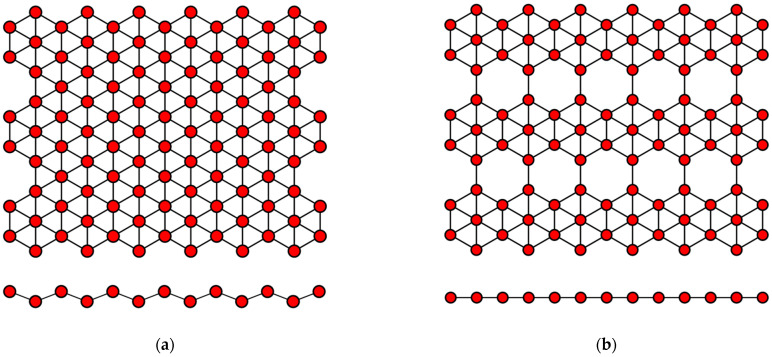
Variants of the structure of the crystal lattice of borophene for (**a**) *v* = 0 and (**b**) *v* = 1/6.

Borophene has a flat or curved structure, consisting of many equilateral triangles, forming a two-dimensional network (Figure 3). In this case, its structure can be considered as a solid solution of the composition B_1−*v*_V*_v_*, where *v* is the concentration of vacancies V of boron atoms. When there are no vacancies of boron atoms, borophene has a curved lattice with the parameter δ ≈ 0.08 nm (Figure 3a), and in the presence of vacancies with the concentration *v* = 1/6, its structure becomes flat (Figure 3b). Borophene structures with a different *v* are also possible. The distance between boron atoms in the borophene structure is about 0.17 nm [2,59].

Gallenene has a flat honeycomb structure similar to graphene (Figure 2a), or a slightly curved configuration such as group IVA transgraphenes (Figure 2b). The crystal lattice parameters of gallenene have the following values: the distance between the gallium atoms is *l* = 0.25 nm, the lattice constant *a* is about 0.39 nm, and the buckling parameter δ = 0–0.08 nm [63,64].

**Figure 4:**
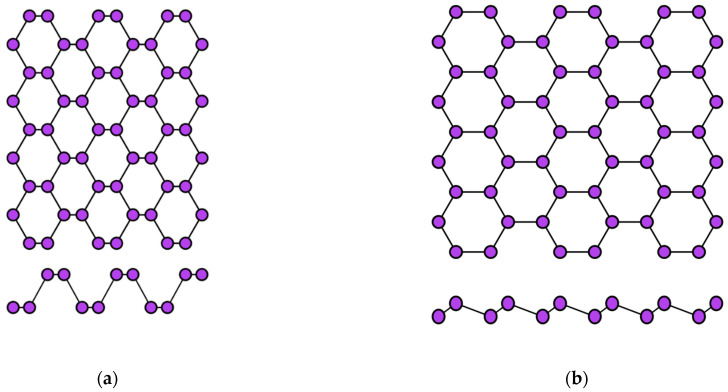
Two variants of the crystal structure of phosphorene: (**a**) puckered (α-phase) and (**b**) buckled (β-phase).

Aluminene has not yet been synthesized experimentally, but theoretical calculations using the density functional theory predict the possibility of its existence with a crystal lattice such as graphene, borophene, or even phosphorene (Figure 4). Thus, aluminene can have a flat, buckled, or puckered structure [67,68]. For the graphene-like modification of the aluminene crystal lattice, the distance between aluminum atoms is predicted to be *l* ≈ 0.26 nm [65].

The two-dimensional allotropic modification of indium (indiene) is also predicted only theoretically so far. Presumably, it can have three variants of the crystal structure: flat (like in graphene) (Figure 2a), buckled (like in transgraphenes of group IVA) (Figure 2b), or puckered (like in phosphorene) (Figure 4a). For all modifications of indiene, the calculated distance between indium atoms is *l* ≈ 0.29 nm, and the lattice constant is *a* ≈ 0.5 nm for the flat geometry and *a* ≈ 0.425 nm for the buckled one at δ ≈ 0.15 nm [65,70,71,72].

The last representative of group IIIA transgraphenes, thallene, was synthesized in 2020 on the NiSi_2_/Si(111) surface [73] in the form of a flat honeycomb structure similar to graphene (Figure 2a), with parameter *l* ≈ 0.38 nm. Density functional theory calculations predict for a free-standing thallene *l* = 0.3 nm (Table 2).

The first representative of transgraphenes of group VA, phosphorene can be in two modifications: puckered (or α-phase) (Figure 4a) and buckled (or β-phase) (Figure 4b). The lattice parameters of phosphorene have the following values: the distance between phosphorus atoms is *l* = 0.23 nm, the lattice constant *a* is about 0.33 nm, and the buckling parameter δ = 0.12 nm [72,74].

For the two-dimensional modification of arsenic (arsenene), theoretical calculations predict the following lattice parameters: the distance between arsenic atoms is *l* = 0.25 nm, the lattice constant *a* is about 0.36 nm, and the buckling parameter δ = 0.14 nm [75,76].

Experimental studies of the synthesis of two-dimensional antimony (antimonene) turned out to be quite successful, and today this material has already been fabricated on a large number of substrates [77,78,79,80,81,82]. The distance between antimony atoms ranges from 0.29 nm and more, depending on the substrate; the lattice constant is slightly more than 0.4 nm and δ ≈ 0.17 nm [75,78].

For bismuthene, a two-dimensional allotropic modification of bismuth, the lattice constant of a curved graphene-like structure was calculated to be *a* = 0.434 nm [72]. However, its actual value may increase depending on the substrate [83] (Table 3).

**Figure 5:**
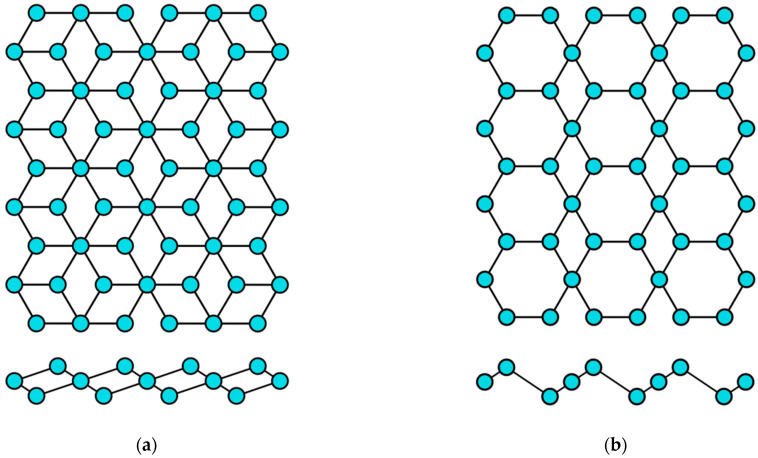
Two variants of the crystal structure of selenene and tellurene: (**a**) α-phase and (**b**) β-phase.

Finally, let us take a look at the structural properties of single-element 2D materials from the group VIA. The stable states of the two-dimensional modification of selenium (selenene) were first calculated within the framework of the density functional theory in 2017 [84]. Then, their electrical, thermoelectric, and thermal properties were theoretically investigated [85,86,87,88]. Two modifications of the crystal lattice are predicted for selenene and tellurene (Figure 5).

According to the theoretical calculations, the distance between atoms in selenene should be *l* ≈ 0.27 nm [88]. The two-dimensional tellurium layer (tellurene) has the same crystal structure modifications as selenene (Figure 5), and its lattice parameter *l* is about 0.3 nm [88,89,90] (Table 4).

Thus, numerous experimental studies confirmed the curved structure of transgraphenes and also showed that such a structure is more flexible than that of graphene, which makes it possible to control the energy spectra by adjusting the synthesis conditions.

## 3. Epitaxial Fabrication of Single-Element 2D Materials

### 3.1. Group IIIA Single-Element 2D Materials

**Figure 6:**
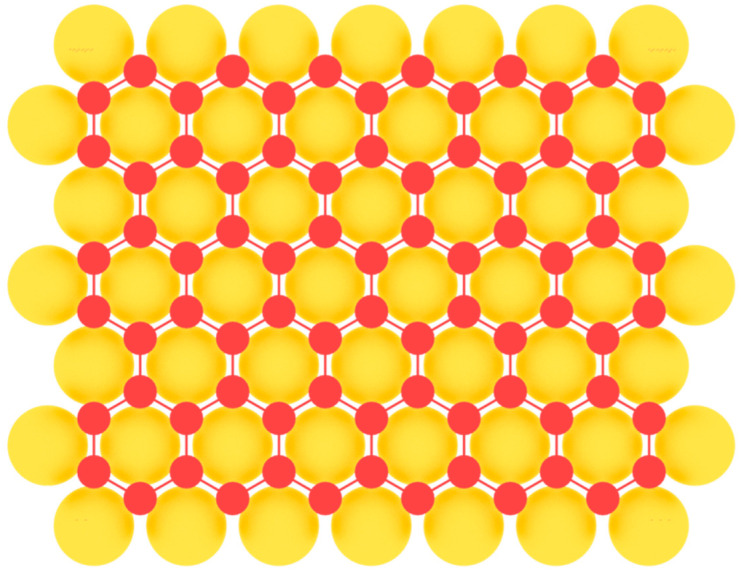
Model of borophene layer on a metal substrate.

We will begin our consideration of two-dimensional allotropic modifications with elements of the main subgroup of the third group of the periodic table of chemical elements. Borophene, the first representative of 2D materials of group IIIA, was first synthesized experimentally on Ag(111) substrate in 2015 [59,60]. Earlier attempts at heteroepitaxial fabrication of borophene were complicated by a low barrier of the formation of three-dimensional boron islands [91]. Therefore, it was necessary to carefully select a suitable substrate in order to overcome this unwanted 3D island formation. A low threshold for nucleation of two-dimensional clusters was found on a number of metal substrates, which facilitated the formation of extended layers of large-area single-crystal borophene. Among them are Al(111) [92], Au(111) [93], Cu(111) [94], and Ir(111) [95] (Figure 6). In this case the nearest-neighbor distance of the potential substrate for the fabrication of 2D material should match with the lattice parameter *a* of the honeycomb lattice. In addition to metal substrates, the possibility of obtaining layers of two-dimensional boron modification on surfaces such as transition metal diborides [96,97] with the (0001) orientation is predicted.

After the first successful experimental works [59,60], borophene was successfully fabricated on the Al(111) surface in 2018 [92]. Aluminum belongs to the same group IIIA of the periodic table and has minimal lattice mismatch with borophene, which ensured its successful production on this substrate. Borophene was deposited on the Al(111) surface held at 230 °C. The obtained borophene had a honeycomb lattice with *a* ≈ 0.3 nm. In another recent study [95], borophene was synthesized on the surface of Ir(111) upon deposition of boron at 550 °C. All experimental studies confirm the possibility of coexistence of several borophene phases depending on the growth conditions and the substrate [91,98,99].

The monatomic layer of two-dimensional gallium was first obtained in 2018 by the exfoliation method in [63]. Like borophene, gallenene was obtained by molecular beam epitaxy on surfaces such as GaN(0001) (two monolayers thick) [100] and Si(111) (one monolayer) [64]. A characteristic feature of gallenene is the covalent bond between adjacent two-dimensional layers, in contrast to other 2D materials, such as, for example, graphite or transition metal dichalcogenides, in which the layers are linked together by weak van der Waals forces. Due to this, the gallium layers demonstrate significantly higher temperature stability [101,102].

In the work [100], two monolayer gallium film with honeycomb lattice was heteroepitaxially grown on GaN(0001) layer with the thickness about 3 µm, which was deposited by metal organic chemical vapor deposition on Al_2_O_3_(0001) substrates using AlN buffer layer. The substrates were chemically cleaned before loading into epitaxy equipment. Several cycles of argon ion sputtering with the energy 700 eV and subsequent annealing were performed to prepare the surface. Two monolayers of gallium were then epitaxially grown at 650 °C from a high-purity gallium (99.995%) source with the deposition rate of 0.4 ML/min (monolayers per minute) at ultra-high vacuum conditions (the base pressure was lower than 2·10^−10^ Torr). This led to formation of 2 ML thick gallenene layer with the lattice constant 0.32 nm [100]. The authors also suggested using silver as capping protective layer for subsequent ex situ characterization of gallenene samples.

In the work [64], base pressure maintained at 1·10^−10^ Torr. Gallium atoms were thermally evaporated on clean Si(111) (7 × 7) surface from a quartz crucible. Reconstructed gallium surface was prepared by depositing 1/3 ML of gallium atoms on the silicon surface at room temperature and annealing at 550 °C for 30 min. The gallium films then were epitaxially grown on the Ga/Si(111) surface at the temperature of 50 °C with the deposition rate of 0.2 ML/min. The gallium layer grown on Si(111) (7 × 7) was used as the substrate for the growth of gallenene. Ga/Si(111) surface is better for obtaining high-ordered gallium monolayer than Si(111) (7 × 7) because gallium atoms passivate the dangling bonds on silicon surface, which is beneficial to the diffusion of the deposited atoms. Depositing Ga atoms on Ga/Si(111) surface, an ordered Ga monolayer can be obtained. At low coverage, the Ga atoms formed two-dimensional domains with random distribution. With the gallium coverage increasing, the size of domains increased. In the area where the gallium atoms were absence, the gallium superstructure was still clearly visible. When the coverage reached 1.4 ML, the Ga/Si(111) reconstructed structure was completely covered, and the growth of the gallium monolayer was completed [64].

Finally, quite recently, there was a report on the heteroepitaxial growth of the last single-element 2D-material of group IIIA, thallene [73]. As far as we know, as of early 2022, this work is the only article dedicated to thallene. The experiment was carried out under ultra-high vacuum conditions. The reconstructed Si(111) (7 × 7) surface was formed by long-term annealing of Si(111) wafers at 600 °C followed by short-term annealing at 1280 °C. One monolayer of thallium was deposited on the prepared surface at room temperature, which was then annealed at the temperature of 300 °C. For thallium evaporation a tantalum crucible was used. The next step was room temperature deposition of one monolayer of nickel, followed by annealing at 300 °C. Under these conditions, nickel atoms diffuse through the thallium layer, forming one monolayer with the composition NiSi_2_ under the thallium layer. The last stage was the annealing of the system at the temperature of 375 °C for 5 min in order to leave 2/3 ML of thallium on the NiSi_2_ surface. As a result, a Tl/NiSi_2_/Si(111) sample with a thallene monolayer was obtained [73].

As far as we know, the rest of the group IIIA transgraphenes (aluminene and indiene) have not been experimentally realized yet.

### 3.2. Group IVA Single-Element 2D Materials

Unlike graphene, a monolayer of carbon atoms with sp^2^-hybridization, which can be fabricated by simple exfoliation of single layers from graphite due to weak interlayer interaction, atom-thick layers of silicon, germanium, tin, or lead are not so readily exfoliated from the corresponding bulk materials because they have sp^3^-hybridization with strong covalent bonds. Consequently, the current task is to find methods of synthesis of transgraphenes other than exfoliation technique. Moreover, one of the basic methods of fabrication of silicene, germanene, stanene, and plumbene today is the heteroepitaxy on the surfaces matched by the lattice parameter [24].

For the first time, silicene was experimentally obtained by epitaxial methods in 2012 on Ag (111) substrate [20,21]. In this work, Ag(111) plates were cleaned by Ar^+^ sputtering with the energy 1.5 keV. Then, the substrates were annealed at the 500–600 °C to obtain atomically smooth silver surface. Silicon was deposited with the rate of 0.01–0.03 ML/min on the silver surface heated to 200–300 °C [20,21]. Later on, research on the growth of silicene on Ag(111) surface at various temperatures was carried out, and the important role of the substrate temperature in the formation of transgraphene structures was established [25]. It was shown that the number of defects in a honeycomb lattice increases with temperature, and silicene does not form at the temperatures of 330 °C or higher.

**Figure 7:**
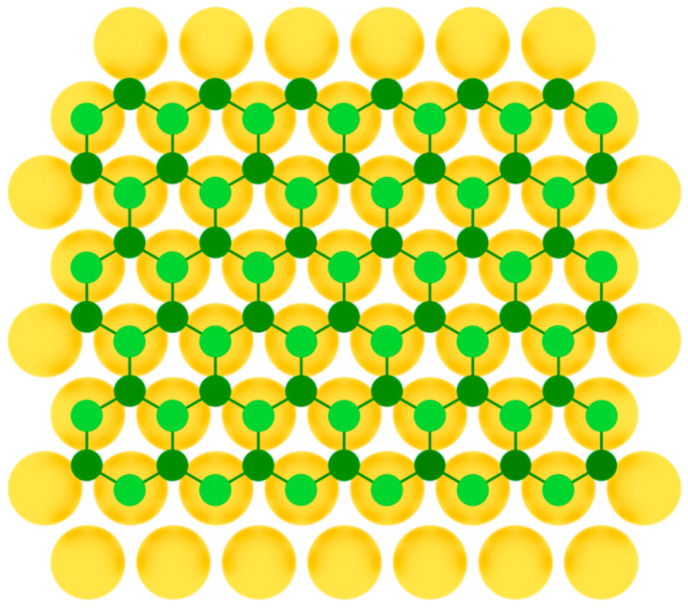
Model of buckled silicene layer on a metal substrate (two colors are used for silicon atoms at different height positions).

After that, silicene was synthesized on Ir(111) [24], Pb(111) [48], ZrB_2_(0001) [26], MoS_2_ [27], ZrC [28], Ru [29], and graphite [30] surfaces. For some substrates, to obtain uniform monolayer with honeycomb lattice, post-growth annealing of the synthesized structures was implemented. As in the previous case of single-element 2D materials of group IIIA, to serve a perfect surface for the group IVA buckled allotropes formation, the substrate should have lattice constant close to that of the desired 2D material, so that three of six atoms in the honeycomb mesh coincide with the nearest three atoms of the substrate (Figure 7).

Unlike silicene, researchers failed to fabricate germanene on Ag(111) surface because germanium and silver atoms form solid solution on the surface. However, thanks to the efforts of experimenters, germanene was finally synthesized on the Au(111) substrate in 2014 [35]. The atomically clean Au(111) surface was prepared by Ar^+^ ion sputtering and annealing. The honeycomb germanene structure was obtained by 1 ML germanium deposition at a temperature of 200 °C. At the same period, germanene was grown on the Pt(111) surface [36]. However, in this case, post-deposition annealing was conducted at the temperatures of 300–500 °C for half an hour.

Then, germanene was heteroepitaxially fabricated on Al(111) substrate [32]. As well as the previously used Au(111) and Pt(111), the Al(111) surface has a honeycomb structure with the lattice parameter *a* = 0.405 nm, which is close to germanene. The germanium deposition at very low growth rate of about 0.005 nm/min was used in this study while keeping the substrate at the temperature of 87 °C.

Later, the growth of germanene was carried out on other surfaces: Sb(111) [49], highly oriented pyrolytic graphite [50,51], MoS_2_ [33], hexagonal AlN [37], Cu(111) [52], Ge_2_Pt [53,54], and germanium [55].

The next representative of group IVA transgraphenes, stanene, was first synthesized on Bi_2_Te_3_(111) surface in 2015 [41]. Bi_2_Te_3_(111) films with thicknesses of up to 40 nm were heteroepitaxially grown on Si(111) substrate. Tin atoms were deposited from effusion cell with the rate of 0.4 ML/min at room temperature on obtained Bi_2_Te_3_(111) surface.

After that, a whole series of works on fabrication of stanene on various surfaces appeared. For example, single-layer stanene was fabricated on Sb(111) and InSb(111) substrates cleaned by Ar^+^ ion sputtering followed by annealing at the 400 °C [42,43]. Tin atoms were deposited with 0.3 ML/min growth rate at ambient or slightly elevated temperatures, forming strained layers of stanene.

Then, layers of large-area stanene with small lattice buckling parameter were obtained on Ag(111) substrate using Ag_2_Sn as an intermediate layer [44]. In one of the latest works [12], high-quality multilayer stanene with a thickness of one to five monolayers was successfully fabricated on PbTe(111) surface. In addition, stanene was fabricated on Cu(111) [56], MoS_2_ [57], and Au(111) [58] surfaces.

Finally, in 2019 the epitaxial fabrication of the last graphene-like material of group IVA, plumbene, was realized [45,46,47]. As far as we know, plumbene has been successfully synthesized on just two surfaces before 2022.

In the research by Yuhara et al. [45], large-area plumbene sheet was fabricated on Pd(111) substrate, cleaned by Ar^+^ ion sputtering with the energy of 2 keV, followed by annealing at 850 °C. Lead deposition rate was maintained at 0.4 ML/min. At the first stage of growth Pd_1−*x*_Pb*_x_*(111) solid solution formed, and then lead atoms segregated onto the surface, forming plumbene structure.

In the latest experimental work [47], plumbene was grown on the surface of Fe monolayer on Ir(111) substrate. To prevent intermixing of iron and lead atoms, the synthesis was performed at rather low temperature of 140 K. The distinctive feature of this method is that an almost flat plumbene monolayer with low buckling parameter forms.

### 3.3. Group VA Single-Element 2D Materials

**Figure 8:**
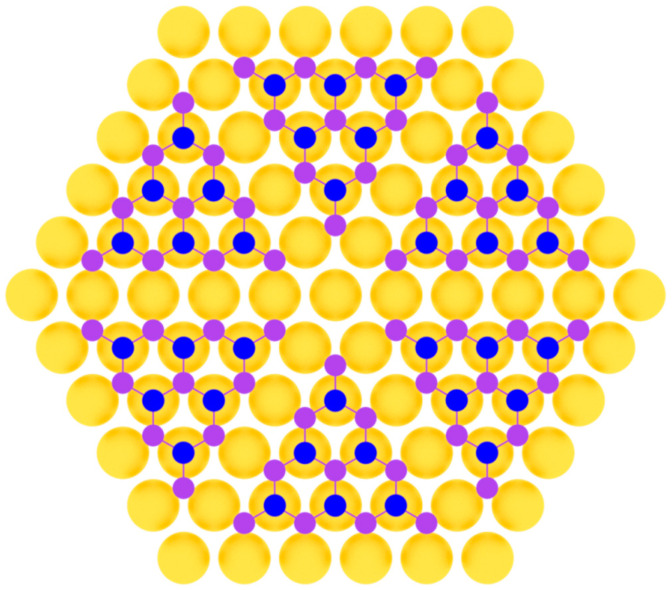
Model of phosphorene layer on a metal substrate (two colors are used for phosphorus atoms at different height positions).

Phosphorene was first produced by an exfoliation technique [103] and later fabricated in the method of molecular beam epitaxy on Au(111) substrate [104]. In work [104], clean Au(111) substrate was prepared by repeated Ar^+^ bombardment with ion energy of 1.5 keV at the pressure of about 1·10^−5^ Torr and subsequent annealing at 500 °C. Phosphorus was deposited by evaporation from a crucible containing bulk black phosphorus at 260 °C. During the deposition process, the substrate temperature was below 260 °C, so the initial growth stage involved the condensation of P_4_ molecules from the gas phase onto Au(111) surface. Then, the system was annealed at 250 °C for 60 min until well-defined monolayer phosphorus with a hexagonal structure appeared [104]. This was achieved due to the close lattice parameters of phosphorene and the substrate (Figure 8).

Later, this result was confirmed in the work [105], along with the observation of formation of one-dimensional chain structures of phosphorus on Au(111) surface. Au(111) substrate was cleaned by Ar^+^ ion sputtering with the energy of 1.0 keV, followed by annealing at a temperature of about 600 °C. Phosphorus flux was generated by thermal decomposition of InP in a Knudsen cell at 470 °C. The substrate temperature was kept at 210–230 °C. As a result, graphene-like structure of phosphorus atoms arranged in a buckled honeycomb lattice was obtained [105].

The electronic structure of this two-dimensional modification of phosphorus was revealed in the further work by Golias et al. [106]. They also prepared clean Au(111) surface by repeated cycles of Ar^+^ bombardment with ion energy 1 keV followed by annealing at 620 °C for 5 min and subsequently at 420 °C for 20 min. Black phosphorus crystal heated to 300 °C was used as a source. Two variants of growth process were tested: one with the Au(111) substrate held at 230 °C during deposition and another where phosphorus was deposited at room temperature, and then the sample was annealed at 250 °C for 15 min.

In the later work by Zhang et al. [107] two distinct superstructures of heteroepitaxial phosphorene on Au(111) were determined with (5 × 5) periodicity (Figure 8). They also used the substrate temperature near the 250 °C, which is line with the authors of previous works. In the very recent work [108], phosphorene was also synthesized on copper oxide surface on Cu(111) substrate using on-surface reaction and segregation approaches.

The first monolayer antimonene samples were obtained by exfoliation techniques [109,110,111,112]. It was shown that free-standing antimonene demonstrates exceptional air stability, but for the creation of defect-free large-area antimonene sheets the epitaxial methods were necessary. Moreover, further experimental attempts were quite successful and now antimonene have been synthesized by the method of molecular beam epitaxy on rather large numbers of substrates, such as Ge(111) [77], PdTe_2_ [78], Ag(111) [79], Pb(111) [80], Bi_2_Te_3_ [81], Cu(111) and Cu(110) [82], and Sb_2_Te_3_ [113]. Furthermore, van der Waals epitaxy was used to form antimonene sheets on mica substrates [114] and single-crystalline graphene [115].

In the work [77], the authors studied the growth of antimonene on Ge(111) surface. Use of Ge(111) surface is prospective for electronics. In these experiments, high-purity Sb crystals were evaporated using a Knudsen cell. The substrate temperature was varied from room temperature to 330 °C, and the deposition rate was changed between 0.2 and 70 nm/min. Ge(111) substrates were prepared firstly by chemical treatment, then by annealing at the temperature of 600–700 °C for more than 1 h. For some samples, an additional Ar^+^ sputtering step with ion energy 2 keV was used before annealing. Sputtering and annealing were repeated until a sharp c(2 × 8) diffraction pattern was observed. After varying growth parameters in wide intervals, it was revealed that 2D antimony sheets were formed in narrow range of temperatures from 200 to 300 °C. For the growth rates it was established that better-quality layers are fabricated using two-step growth with the nucleation of antimony clusters at high rates (about 20 nm/min) and subsequent growth of islands at much lower rates. It was also shown that the lattice constant of antimonene depends on the thickness and changes from 0.41 nm for one layer to 0.43 nm for five layers [77].

Authors of the work [78] noted that they selected PdTe2 substrate for the synthesis of antimonene. This selection was made because PdTe2 substrate has hexagonal lattice with the constant of 0.41 nm (close to the calculated one for antimonene), along with the chemical stability of PdTe_2_ surface and absence of formation of an alloy with antimony. High-purity antimony was evaporated from Knudsen cell and deposited onto the freshly cleaved from the single crystal PdTe_2_ substrate. The substrate temperature was held at 130 °C during growth. This deposition resulted in formation of highly ordered buckled honeycomb antimonene structure [78].

Despite the fact that antimony tends to form alloys with metals, some groups reported the fabrication of antimonene on silver [79,116] and copper [82] substrates. In all cases, the first atoms of antimony formed an alloy with the substrate, and subsequent deposition led to the formation of buckled honeycomb antimonene structure. However, all the prospective properties of antimonene are still not realized due to insufficient quality of the fabricated antimonene, especially in the areas of edges of 2D sheets.

As far as the arsenene is concerned, there are numerous theoretical works devoted to the prediction of properties of this novel 2D material [75]. However, there is only one experimental report on the fabrication of arsenene by liquid-phase exfoliation method [76].

The same is true for bismuthene, and there are just a couple of experimental evidences of its fabrication [83,117]. In the breakthrough work [83], bismuthene was fabricated on SiC(0001) surface. Moreover, in the very latest work [117], two-dimensional hexagonal bismuth structures were obtained on the surface of 2 ML thick HfTe_2_ surface deposited on InAs(111)/Si(111) substrate. Furthermore, the attempts to obtain bismuthene on other surfaces (such as Si(111), graphite, graphene, Au(111), and other noble metals) are constantly being made.

### 3.4. Group VIA Single-Element 2D Materials

To date, selenene was synthesized only by physical vapor deposition on Si(111) substrate in the work [118].

**Figure 9:**
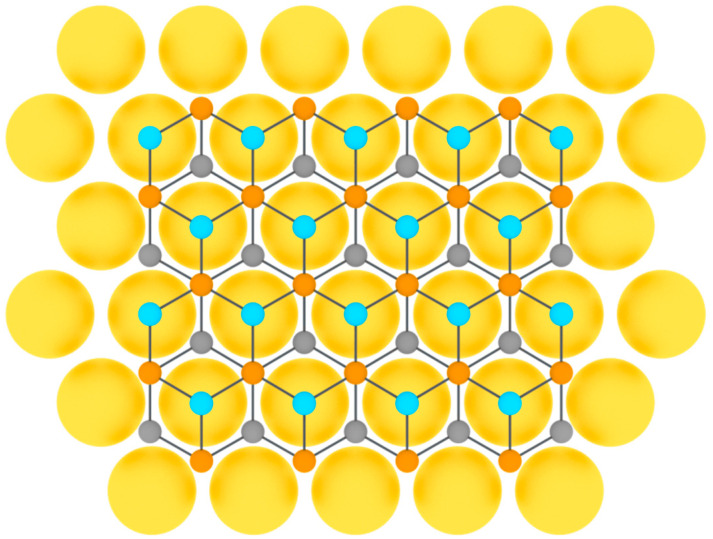
Model of tellurene layer on a substrate (three colors are used for tellurium atoms at different height positions).

Tellurene was first obtained on flexible mica substrates by van der Waals epitaxy in 2014 in [119]. Thereafter, tellurium was successfully grown heteroepitaxially on highly oriented pyrolytic graphite [89] and graphene [120] (Figure 9).

In the work [120], different phases of two-dimensional tellurene were obtained in monolayer and few-layer films using van der Waals epitaxy of tellurium films on the surface of graphene, grown by molecular beam epitaxy on 6H-SiC(0001) substrate. High-purity tellurium (99.999%) was evaporated from a Knudsen cell at 227 °C onto the graphene/SiC substrate kept at room temperature.

In [89], tellurium atoms were deposited on a carefully annealed substrate of highly oriented pyrolytic graphite under the conditions of a molecular beam epitaxy setup at the base pressure of order of 1.5 10^–10^ Torr. The flux of tellurium was generated from a standard Knudsen cell operated at 270 °C. The substrate temperature was held constant at 130 °C during deposition. Reflection high-energy electron diffraction operated at 15 keV was employed to monitor the sample surface during the growth process, and the streaky diffraction pattern showed an atomically smooth surface of the grown tellurium film, which was confirmed by STM measurements [89].

Thus, a proper substrate and growth conditions play a key role in providing an opportunity to form large-size high-quality single-element two-dimensional materials beyond graphene.

## 4. Brief Outlook into the Perspectives of Single-Element 2D Materials

At present, two-dimensional materials are considered as one of the most promising materials for next-generation nanoelectronics and nanophotonics [121,122]. The family of two-dimensional crystals includes dielectrics (for example, hexagonal boron nitride and transition metal oxides), topological insulators (bismuthene telluride), semiconductors (molybdenum and tungsten disulfides), semimetals (graphene), metals (titanium disulfide), and superconductors (niobium diselenide) [3,4]. Among the single-element transgraphenes considered in this review, the type of conductivity changes from metal (for group IIIA elements), passes through semimetal (for group IVA elements), and ends with groups VA and VIA semiconductor materials (Table 5). This fact determines the enormous spectrum of possible device applications of single-element 2D materials.

For example, a high-speed field-effect transistor based on silicene operating at room temperature has already been implemented [8], as well as a field-effect transistor based on tellurene [123]. Among other things, tellurene also possesses unique thermoelectric properties [124] and anomalously low thermal conductivity [125,126,127]. In 2D materials such as stanene and bismuthene, due to their large atomic number, nontrivial topological properties are expected, the appearance of highly conductive channels, and the absence of certain types of carrier scattering near the boundaries. In this regard, they can be considered contenders for the creation of effective interconnections [9,128]. Aluminene and antimonene are proposed as materials for creating nanocapacitors due to their high ability to accumulate the charge [129]. In addition, superconducting states are also predicted for aluminene and gallenene [130,131]. Possible spheres of single-element 2D materials applications are summarized in Table 5.

Active research continues, aimed at finding new and investigating the discovered mechanical, electrical, magnetic, and optical properties of single-element 2D materials [132,133,134,135,136,137,138,139]. Fascinating novel physical phenomena are also theoretically predicted for materials of this family that are still waiting to be experimentally discovered and investigated. Among them are various plasmonic effects and quantum Hall effect in 2D boron [91], high-temperature quantum spin Hall effect in bismuthene [83], strain-induced magnetism in aluminene [140], massless Dirac fermions, and superconductivity in gallenene [131].

Most of the papers reviewed in this work are aimed at developing methods for the creation of high-quality layers of single-element 2D materials, which make it possible to study all of their unique physical properties and potential applications in emerging nanodevices of functional electronics. Undoubtedly, the fabrication of 2D materials of the required quality dictates a proper selection of the surface for synthesis (according to the crystal structure and lattice constant of the growing material). The next stage is rigorous preparation of the growth substrate using multiple ion sputtering and high-temperature annealing to obtain an atomically smooth layer without impurities and defects. The final step is precise control of the growth conditions, such as deposition temperature (usually less than 500 °C) and extremely low growth rates (on the order of 0.1 mL/min). The coverages of the deposited materials should be mostly chosen to equal the whole number of monolayers and no more than about 5 mL because the growth mechanism tends to transform to the island one at higher effective thicknesses.

Extensive amount of research is underway to improve the technology for growing high quality 2D crystals [141,142,143,144,145]. Physical and mathematical models of growth of 2D materials by various growth mechanisms, taking into account formation of 2D and 3D islands, as well as ways to prevent possible nucleation of unwilling three-dimensional islands, are proposed [146,147,148,149]. There are also theoretical and experimental works devoted to various derivatives of transgraphenes, including chemical functionalization with various ligands [150,151,152].

Completely new, unexpected fields of application of transgraphenes and their derivatives are being proposed [153,154,155,156,157,158]. For instance, a material such as borophene is predicted to serve as an effective energy storage and also may form nanotubes analogous to those of graphene, which may have even higher electrical conductivity that suggests the possibility of their use as contacts and interconnections in nanodevices [91]. Multi-layer gallenene structures are expected to have significantly higher temperature stability than bulk material [102]. Strained layers of stanene have potential as possible room-temperature device applications due to a very wide bandgap induced by elastic strains [42,43]. Plumbene is calculated to have controllable sign reversal Seebeck coefficient and the large tunability of thermal conductivity [159]. An opportunity to manage band gap in wide ranges was experimentally established for antimonene [112].

In order to realize all the promising properties of single-element 2D allotropes, one needs to understand how to create defect-free large-area single-crystal structures and multilayer 2D materials. Many of the prospective properties of these materials are still not realized due to insufficient quality of the fabricated sheets. Therefore, careful preparation of the substrate and the creation of special conditions in the epitaxy chamber are required for the preferred formation of islands by the two-dimensional mechanism at the initial stages of epitaxial growth. In this regard, molecular beam epitaxy has a major advantage compared to other fabrication techniques. Epitaxial methods usually yield large-scale 2D sheets with the area limited mainly by the width of atomic terraces on the substrate. Moreover, thorough pre-epitaxial preparation of the substrate ensures uniform two-dimensional wetting of the atomically flat surface by depositing atoms with limited nucleation of unwilling volumetric clusters [128]. Nevertheless, the problem of co-existence of differently rotated 2D grains with various phases in such large-area structures is still not solved. Hence, it is necessary to comprehensively develop the technology of epitaxial synthesis of 2D materials. Thus, the successful fabrication of graphene-like materials is possible only with the correct choice of the growth surface, its careful preparation, and precise control of the growth conditions [160,161].

Besides molecular beam epitaxy, there are some other methods of producing single-element 2D materials. The basic technologies among them are exfoliation techniques (including mechanical [109], liquid-phase [162], and molten salt exfoliation [163]), chemical vapor deposition [164], and physical vapor deposition [118]. Moreover, some less-used methods can be mentioned, such as pulsed laser deposition [165] and atomic layer deposition [166]. The important challenge for the research groups among the world is to develop all these methods simultaneously in order to produce 2D nanostructures of better quality.

After the synthesis of transgraphene, researchers are faced with the huge (and mostly unsolved) problem of separation of 2D sheets or transferring of this 2D material from metallic to other device-ready substrates. Moreover, single-element two-dimensional transgraphenes are dramatically unstable when exposed to the air, resulting in their rapid oxidation. This issue demands a decision of questions connected with encapsulation of fabricated layers to prevent them from chemical destruction. Along with the task of controlled fabrication of large-scale high-quality samples, these are the three pivotal limitations that hinder further characterization and device applications of single-element transgraphenes.

Fabrication of remaining transgraphenes (aluminene, indiene, and possible two-dimensional allotropes of heavier elements) is also strongly anticipated to complete the puzzle of synthesized single-element 2D materials in the periodic table.

The star of single-element two-dimensional materials lit up very recently, and it has strong potential that could really shine in the very near future.

## Figures and Tables

**Table 1:** Structural parameters of the buckled honeycomb lattice of graphene and group IVA transgraphenes.

Material	Distance *l* between Atoms	Lattice Constant *a*	Buckling Parameter δ	References
	nm	nm	nm	
Graphene	0.14	0.25	0	[3,4,5]
Silicene	0.23	0.39	0.08	[20,21,22,23,24,25,26,27,28,29,30,31]
Germanene	0.25	0.40	0.09	[32,33,34,35,36,37]
Stanene	0.28	0.47	0.10	[38,39,40,41,42,43,44]
Plumbene	0.30	0.49	0.10	[45,46,47]

**Table 2:** Parameters of the honeycomb lattice of group IIIA transgraphenes (materials predicted only theoretically and not obtained experimentally are marked with * sign).

Material	Distance *l* between Atoms	Lattice Constant *a*	Buckling Parameter δ	References
	nm	nm	nm	
Borophene	0.17	0.29	0–0.08	[2,59]
Aluminene *	0.26	0.45	-	[65]
Gallenene	0.25	0.39	0–0.08	[63,64]
Indiene *	0.29	0.43–0.50	0–0.15	[65,70,71,72]
Thallene	0.30–0.38	0.50–0.65	0	[73]

**Table 3:** Parameters of the honeycomb lattice of group VA transgraphenes.

Material	Distance *l* between Atoms	Lattice Constant *a*	Buckling Parameter δ	References
	nm	Nm	nm	
Phosphorene	0.23	0.33	0.12	[72,74]
Arsenene	0.25	0.36	0.14	[75,76]
Antimonene	0.29	0.40	0.17	[75,78]
Bismuthene	0.30	0.43	0.17	[72,83]

**Table 4:** Parameters of the honeycomb lattice of group VIA transgraphenes.

Material	Distance *l* between Atoms	Lattice Constant *a*	Buckling Parameter δ	References
	nm	nm	nm	
Selenene	0.27	0.37	0.18	[88]
Tellurene	0.30	0.42	0.22	[88,89,90]

**Table 5:** Physical properties and application fields of transgraphenes (materials predicted only theoretically and not obtained experimentally are marked with * sign).

Material	Band Gap, eV	Type of Conductivity	Possible Fields of Application
Borophene	0	Metal	bio-imaging tools, microelectronics devices, composites, interconnections, energy storage
Aluminene *	0	Metal	nanocapacitor, superconductor, gas sensor, energy storage
Gallenene	0	Metal	superconductor, electrical contacts, sensors, plasmonics, photonics nanostructures
Indiene * (planar) Indiene * (buckled)	0 1	Metal Semiconductor	memory device, LED, solar cell, light filter, optoelectronics applications
Thallene	0.14	Semimetal	2D topological insulator
Graphene	0	Semimetal	field-effect transistor, phototransistor, superconductor, optical modulator, plasmonics, photovoltaics applications
Silicene	0.01	Semimetal	field-effect transistor, biosensor, spintronics, plasmonics, quantum information applications
Germanene	0.02	Semimetal	field-effect transistor, nanomedicine, topological quantum field-effect transistor
Stanene	0.07	Semimetal	2D topological insulator, superconductor, field-effect transistor, interconnections
Plumbene	0.42	Semiconductor	2D topological insulator, superconductor, field-effect transistor, energy storage
Phosphorene (puckered) Phosphorene (buckled)	1.67 1.98	Semiconductor	field-effect transistor, phototransistor, biosensor, bio-imaging tools
Arsenene (puckered) Arsenene (buckled)	0.90 1.96	Semiconductor	field-effect transistor, biosensor
Antimonene (puckered) Antimonene (buckled)	0.28 0.76	Semiconductor	field-effect transistor, nanocapacitor, photodetector, sensor, bio-imaging tools
Bismuthene	0.32	Semiconductor	2D topological insulator, field-effect transistor, biosensor, bio-imaging tools, interconnections
Selenene	0.75	Semiconductor	field-effect transistor, phototransistor
Tellurene	1.13	Semiconductor	field-effect transistor, optical modulator, thermoelectric material, piezoelectric material

## Data Availability

The authors declare that the data supporting the findings of this study are available within the article.
